# Targeting CHAF1B Enhances IFN Activity against Myeloproliferative Neoplasm Cells

**DOI:** 10.1158/2767-9764.CRC-23-0010

**Published:** 2023-05-31

**Authors:** Diana Saleiro, Ewa M. Kosciuczuk, Mariafausta Fischietti, Ricardo E. Perez, G. Sohae Yang, Frank Eckerdt, Elspeth M. Beauchamp, Ye Hou, Qixuan Wang, Rona Singer Weinberg, Eleanor N. Fish, Feng Yue, Ronald Hoffman, Leonidas C. Platanias

**Affiliations:** 1 Robert H. Lurie Comprehensive Cancer Center of Northwestern University, Chicago, Illinois.; 2 Division of Hematology-Oncology, Department of Medicine, Feinberg School of Medicine, Northwestern University, Chicago, Illinois.; 3 Department of Medicine, Jesse Brown Veterans Affairs Medical Center, Chicago, Illinois.; 4 Department of Biochemistry and Molecular Genetics, Feinberg School of Medicine Northwestern University, Chicago, Illinois.; 5 The New York Blood Center, New York, New York.; 6 Myeloproliferative Neoplasms Research Consortium, New York, New York.; 7 Toronto General Hospital Research Institute, University Health Network & Department of Immunology, University of Toronto, Toronto, Ontario, Canada.; 8 Tisch Cancer Institute, Icahn School of Medicine at Mount Sinai, New York, New York.

## Abstract

Interferons (IFNs) are cytokines with potent antineoplastic and antiviral properties. IFNα has significant clinical activity in the treatment of myeloproliferative neoplasms (MPN), but the precise mechanisms by which it acts are not well understood. Here, we demonstrate that chromatin assembly factor 1 subunit B (CHAF1B), an Unc-51-like kinase 1 (ULK1)-interactive protein in the nuclear compartment of malignant cells, is overexpressed in patients with MPN. Remarkably, targeted silencing of *CHAF1B* enhances transcription of IFNα-stimulated genes and promotes IFNα-dependent antineoplastic responses in primary MPN progenitor cells. Taken together, our findings indicate that CHAF1B is a promising newly identified therapeutic target in MPN and that CHAF1B inhibition in combination with IFNα therapy might offer a novel strategy for treating patients with MPN.

**Significance:**

Our findings raise the potential for clinical development of drugs targeting CHAF1B to enhance IFN antitumor responses in the treatment of patients with MPN and should have important clinical translational implications for the treatment of MPN and possibly in other malignancies.

## Introduction

Myeloproliferative neoplasms (MPN) are a group of hematologic malignancies characterized by aberrant myeloid cell proliferation and increased risk of transformation to acute myeloid leukemia (1, 2). There are three types of MPN: polycythemia vera (PV), essential thrombocythemia (ET), and primary myelofibrosis (PMF; refs. 3, 4). The somatic mutations that drive the development of MPN phenotypes occur in hematopoietic stem cells, the most common being the substitution of a valine for a phenylalanine at the 617 position in the Janus kinase 2 gene (*JAK2*^V617F^), an intracellular tyrosine kinase, which is activated downstream of the interaction between hematopoietic growth factors and their receptor (4–6). In addition, somatic mutations in *CALR* (calreticulin)*, MPL* (MPL proto-oncogene, thrombopoietin receptor), and *TET2* (Tet methylcytosine dioxygenase 2) genes are also detected in patients with MPN (4).

Interferon-alpha (IFNα) is currently one of the drugs considered the standard of care for patients with PV and ET (7–10). Type I IFNs bind to specific cell-surface IFN receptors activating distinct signaling pathways that mediate the transcription and translation of IFN-stimulated genes (ISG), which ultimately account for their unique IFN biological effects (11–14). Although there have been substantial advances in our understanding of the IFN signaling, the connection and specificity of signaling events that correlate with clinical responses in MPN remains to be defined.

We have previously shown that Unc-51-like kinase 1 (ULK1) plays a central and essential role in the generation of IFN responses in PV (15, 16). In the current study, we identify chromatin assembly factor 1 subunit B (CHAF1B) as a novel negative regulator of IFNα-inducible ULK1-driven signaling pathways in MPN cells that may control IFNα-mediated anti-MPN responses. We demonstrate that CHAF1B, a protein component of the chromatin assembly factor 1 complex (CAF-1; ref. 17), is overexpressed in the peripheral blood (PB) of patients with MPN and interacts with ULK1 in the nuclear compartment. Disruption of *CHAF1B* expression strongly enhances the effects of IFNα on MPN cells. Our findings raise the possibility of clinical development of CHAF1B inhibitors to enhance IFNα-driven antitumor responses in MPN and possibly additional IFN-responsive malignancies.

## Materials and Methods

### Cells, Cell Culture, and Reagents

HEL cells (#TIB-180; ATCC; RRID:CVCL_2481) were cultured in RPMI1640 medium (Gibco) supplemented with 10% FBS and antibiotics. SET-2 cells (#ACC 608; DSMZ; RRID:CVCL_2187) were cultured in RPMI1640 medium (Gibco) supplemented with 20% FBS and antibiotics. All cell lines were frozen at low passage in liquid nitrogen and were kept in culture for no longer than 15 passages. All cells were routinely tested for *Mycoplasma* contamination using a MycoAlert PLUS *Mycoplasma* detection kit, following the manufacturer's instructions (Lonza). Cell lines were authenticated by short tandem repeat analyses and matched to the ATCC or DSMZ database every 6 months to 1 year.

PB from PV patients was collected after obtaining written informed consent approved by the Institutional Review Board of Northwestern University (Chicago, IL) and PB mononuclear cells (MC) were isolated following Histopaque density gradient separation (Sigma). PBMCs or bone marrow (BM) MCs from PV patients were also obtained from the New York Blood Center bank. These samples were collected from patients with PV who participated in the Myeloproliferative Disorders Research Consortium (MPD-RC-111) study, after obtaining patients’ written informed consent and approved by the respective Institutional Review Boards of Institutions participating in the MPN Research Consortium. The MPD-RC-111 study was an investigator-initiated, international, multicenter, phase II trial evaluating the ability of pegylated-rIFN-α2a therapy to induce complete and partial hematologic responses in patients with high-risk ET or PV who were either refractory or intolerant to hydroxyurea (clinical trial #NCT01259817). PBMCs or BMMCs were collected at baseline, prior to IFN treatment. The criteria for enrollment of patients and a detailed description of participants enrolled in clinical trial #NCT01259817 have been described previously (18).

ON-TARGETplus non-targeting control pool siRNAs (#D-001810-10-05) and human *CHAF1B* SMARTPool siRNAs (#L-019937-00-0005) were purchased from Horizon Discovery (Dharmacon). Control and *ULK1* targeting siRNAs were obtained from Santa Cruz Biotechnology (control siRNA-H #sc-44236 and human ULK1 siRNA #sc-44182). Anti-CHAF1B antibody (WB – 1:1,000, ChIP: 2 μg/IP; #27633-1-AP, RRID:AB_2880933) was from Proteintech. Anti-ULK1 (WB – 1:1,000, co-IP – 2.1 μg/mg protein lysate; #8054, RRID:AB_11178668) and anti-lamin A/C (WB – 1:1,000, #2032, RRID:AB_2136278) antibodies were from Cell Signaling Technology and anti-α-tubulin antibody (WB – 1:1,000, #sc-5286, RRID:AB_628411) was from Santa Cruz Biotechnology. Anti-GAPDH antibody (WB – 1:20,000, #MAB374, RRID:AB_2107445) was from EMD Millipore. The ULK1 inhibitor SBI-0206965 (SBI) was purchased from Cayman Chemical (# 18477).

### Nano-LC/MS-MS Analysis

To identify new binding partners for endogenous ULK1, HEL cells were either left untreated or were treated with recombinant human interferon alfacon-1 (IFNα; 5 × 10^3^ IU/mL) for 4 hours, then lysed to isolate cytoplasmic and nuclear proteins using the NE-PER Nuclear and Cytoplasmic Extraction Reagents (Thermo Fisher Scientific, #78833) according to the manufacturer's protocol. Three milligrams of cytoplasmic and nuclear cell protein lysates, respectively from untreated and IFNα-treated samples were used for immunoprecipitation (IP) of endogenous ULK1 complexes using ULK1 (D8H5) rabbit mAb (2.1 μg/mg protein; Cell Signaling Technology, #8054, RRID:AB_11178668). As control, the same procedure was followed for IFNα-treated lysates but using rabbit (DA1E) mAb IgG XP isotype control (Cell Signaling Technology, #3900, RRID:AB_1550038) instead of the ULK1 antibody. After incubating the samples with antibody overnight with rotation at 4°C, these were incubated with protein G sepharose 4 Fast Flow beads (GE Healthcare) for 1 hour with rotation at 4°C, then washed two times with NP-40 buffer [20 mmol/L Hepes (pH 7.4), 180 mmol/L KCl, and 0.2 mmol/L Ethyleneglycol-bis(β-aminoethyl)-N,N,Nʹ,Nʹ-tetraacetic Acid (EGTA), 0.1% NP-40] and one time with washing buffer [20 mmol/L Hepes (pH 7.4), 180 mmol/L KCl, and 0.2 mmol/L EGTA]. Protein–ULK1 complexes were eluted from the beads by incubation with Lane Marker Reducing Sample Buffer (Pierce) at 95°C for 10 minutes, and proteomic analyses were performed by the Northwestern Proteomics Core Facility (Northwestern University, Chicago, IL), as in previous studies (16, 19).

### Protein Function Enrichment Analysis

Protein lists identified in nano-liquid chromatography tandem mass spectrometry analysis (nLC/MS-MS), before and after IFNα treatment, in cytosolic and nuclear fractions were converted to gene lists that were submitted to the Metascape database (http://metascape.org; RRID:SCR_016620) for pathway and process enrichment analysis (20), as in our previous studies (19).

### co-IP and Immunoblotting

HEL and SET-2 cells were either left untreated or were treated with IFNα (5 × 10^3^ IU/mL) for either 10 minutes or 4 hours, as indicated. Cells were lysed to isolate cytoplasmic and nuclear proteins using the NE-PER Nuclear and Cytoplasmic Extraction Reagents, according to the manufacturer's protocol. A total of 700 μg of protein lysates from cytoplasmic and nuclear fractions were used for IP of endogenous protein–ULK1 complexes and were incubated overnight at 4°C with rotation with ULK1 (D8H5) rabbit mAb (2.1 μg/mg protein; Cell Signaling Technology, #8054, RRID:AB_11178668), followed by incubation for 1 hour at 4°C with rotation with protein G Sepharose 4 Fast Flow beads (GE Healthcare). As control, the same procedure was followed, using nonimmune rabbit IgG XP Isotype control antibody (DA1E; Cell Signaling Technology, #3900, RRID:AB_1550038) instead of anti-ULK1 antibody. After the incubation, the beads were washed three times with NP-40 buffer [20 mmol/L HEPES (pH 7.4), 180 mmol/L KCl, 0.2 mmol/L EGTA, and 0.1% NP-40]. Protein–ULK1 complexes were eluted from the beads by incubation with Lane Marker Reducing Sample Buffer (Pierce) at 95°C for 10 minutes. Eluates were resolved by SDS-PAGE and transferred to an Immobilon-P polyvinylidene difluoride (PVDF) membrane (Millipore). For immunoblotting analyses, the membranes were then probed with primary antibodies, followed by horseradish peroxidase–conjugated secondary antibodies, and antibody binding was detected by enhanced chemiluminescence using Amersham Biosciences ECL Prime Western blotting detection reagent (GE Healthcare Life Sciences), as in our previous studies (16, 19).

### *CHAF1B* Expression in Patients with MPN

log_2_ of transcript levels of *CHAF1B* in neutrophils from patients with MPN with PV, ET, and PMF compared with healthy individuals was calculated using GSE54646 dataset (21) accessible through NCBI Gene Expression Omnibus (GEO; RRID:SCR_005012) database (22). In addition, using the mutational status information available for the same cohort of patients with MPN, we compared log_2_ of *CHAF1B* levels in patients carrying wild-type versus mutant *JAK2*, *CALR, TET2,* and *MPL* genes.

### qRT-PCR

HEL and SET-2 cells were transfected with either control siRNA or siRNAs targeting *CHAF1B* using Amaxa Biosystems Nucleofector Kit V and program X-005 for HEL cells and X-013 for SET-2 cells (Lonza) per the manufacturer's instructions*.* After 48 hours, transfected cells were either left untreated or were treated with IFNα (5 × 10^3^ IU/mL) for 6 hours. RNA was isolated using the RNeasy Mini Kit (Qiagen) per the manufacturer's instructions. A total of 2 μg of total cellular mRNA was reverse transcribed into cDNA using the OmniScript RT kit (Qiagen) and oligo(dT)12–18 primers (Life Technologies). qRT-PCR was carried out using the Bio-Rad CFX96 Real-Time System machine (Bio-Rad) using commercially available FAM-labeled probes and primers (Thermo Fisher Scientific) to determine the expression of human *CHAF1B* (Hs01123302_m1), *ISG15* (Hs01921425_s1), *MX1* (Hs00895608_m1), *IFIT1* (Hs00356631_g1), and *IRF7* (Hs01014809_g1). Human *GAPDH* (Hs02758991_g1) was used for normalization. Relative quantitation of mRNA levels was calculated using the ΔΔCt method and plotted as fold increase compared with control siRNA-transfected untreated cells.

### Chromatin Immunoprecipitation Assay

Chromatin immunoprecipitation (ChIP) assays were performed as reported previously (23) using untreated versus IFNα-treated HEL cells, untreated versus IFNα-treated control siRNA and *ULK1* siRNA-transfected HEL cells, and vehicle (DMSO), SBI-0206965 (10 μmol/L) and/or IFNα-treated HEL cells, as indicated in the respective figure legends, and using the SimpleChIP Enzymatic Chromatin IP Kit with Magnetic Beads (#9003, Cell Signaling Technology) per manufacturer's instructions. HEL cells were transfected with either control siRNA or siRNAs targeting *ULK1* using Amaxa Biosystems Nucleofector Kit V and program X-005 (Lonza) per the manufacturer's instructions one day prior to IFNα treatment. Anti-CHAF1B antibody (Proteintech, #27633-1-AP, RRID:AB_2880933) was used for IP of CHAF1B, and normal rabbit IgG (Cell Signaling Technology, #2729, RRID:AB_1031062) was used as negative control. qRT-PCR was performed on purified immunoprecipitated DNA for the *ISG15 and IFIT1* promoter binding sites using SsoAdvanced Universal SYBR Green supermix (Bio-Rad) following manufacturer's guidelines with the following primers: *ISG15* FWD 5′-CCA CTT TTG CTT TTC CCT GTC-3′, *ISG15* REV 5′-AGT TTC GGT TTC CCT TTC CC-3′ and *IFIT1* FWD 5′-GGT TGC AGG TCT GCA GTT TAT CTG T-3′ and *IFIT1* REV 5′-AGC TGT GGG TGT GTC CTT GC-3′. All qRT-PCR signals were normalized to the input DNA.

### Hematopoietic Cell Progenitor Assays

In the experiments to assess the role of CHAF1B in the inhibitory effects of IFNα-mediated anticlonogenic effects, HEL and SET-2 cells were transfected with either control siRNA or siRNAs targeting *CHAF1B* using Amaxa Biosystems Nucleofector Kit V and program X-005 for HEL cells and X-013 for SET-2 cells (Lonza) per the manufacturer's instructions. Twenty-four hours later, control siRNA or *CHAF1B* siRNA-transfected cells were plated in MethoCult H4534 Classic medium (#04534, StemCell Technologies) and were either left untreated or were treated with IFNα (100 IU/mL for HEL and 50 IU/mL for SET-2). Leukemic colony-forming units (CFU-L) formation was assessed as in our previous studies (15, 16, 24). In addition, primary PBMCs or BMMCs from patients with PV were transfected with either control siRNA or *CHAF1B* siRNAs using *Trans*IT-TKO Transfection Reagent (Mirus) and were either left untreated or were treated with IFNα (1,000 IU/mL). Human erythroid progenitor cells [burst-forming unit erythroid (BFU-E)] were assayed in MethoCult H4434 Classic medium (#04434, StemCell Technologies), as described in our previous studies (15, 16). Data are expressed as the percentage of colony formation over untreated control siRNA-transfected cells.

### Statistical Analyses

All statistical analyses were performed using GraphPad Prism 8.0 (RRID:SCR_002798). Statistical tests used are described in each figure legend. *P* values less than 0.05 were considered statistically significant.

### Data Availability

The original data that support the findings of this study are available from the corresponding author upon reasonable request. The mass spectrometry proteomics data generated in this study have been deposited to the ProteomeXchange Consortium (RRID:SCR_004055) via the PRIDE partner (25) repository with the dataset identifier PXD037376.

## Results

### Proteomic Screening Identifies CHAF1B as a Novel Binding Partner of ULK1

**FIGURE 1:**
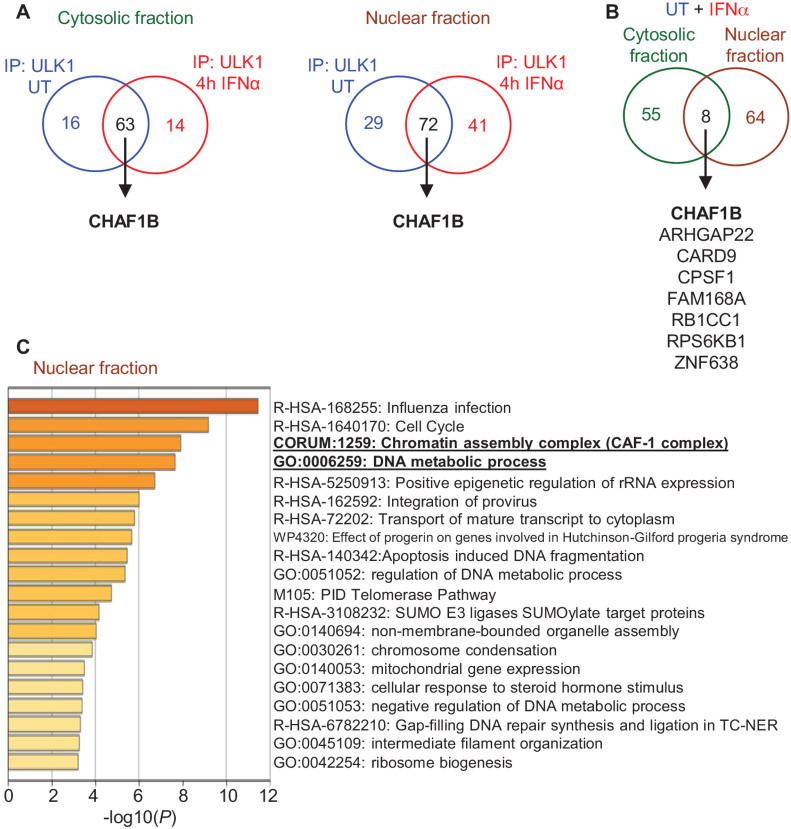
Mass spectrometry identification of cytosolic and nuclear putative binding partners of ULK1. **A** and **B,** Endogenous ULK1–protein complexes were immunoprecipitated (IP) using an anti-ULK1 antibody from cytosolic and nuclear fractions isolated from untreated (UT) or IFNα-treated [4 hours (4h)] HEL cells and analyzed by nLC/MS-MS. As negative control, IP with normal rabbit IgG was used instead of anti-ULK1 antibody and proteins bound to rabbit IgG were excluded from the analyses. **A,** Venn diagrams show the number of putative ULK1 binding partners identified only in untreated cells (blue), only in IFNα-treated cells (red), or both treated and untreated cells (black). CHAF1B was detected in both cell compartments by mass spectrometry. **B,** Venn diagram shows the overlap for the putative ULK1 binding partners identified under both untreated and IFNα-treated cells in the cytosolic and nuclear fractions. List of the putative ULK1 binding proteins found under these conditions on both cytosolic and nuclear compartments is shown. **C,** Gene ontology analyses of the 72 putative nuclear ULK1-interacting proteins identified under both untreated and IFNα-treated conditions are shown. Underlined and in bold are the biological events in which CHAF1B is involved.

**FIGURE 2:**
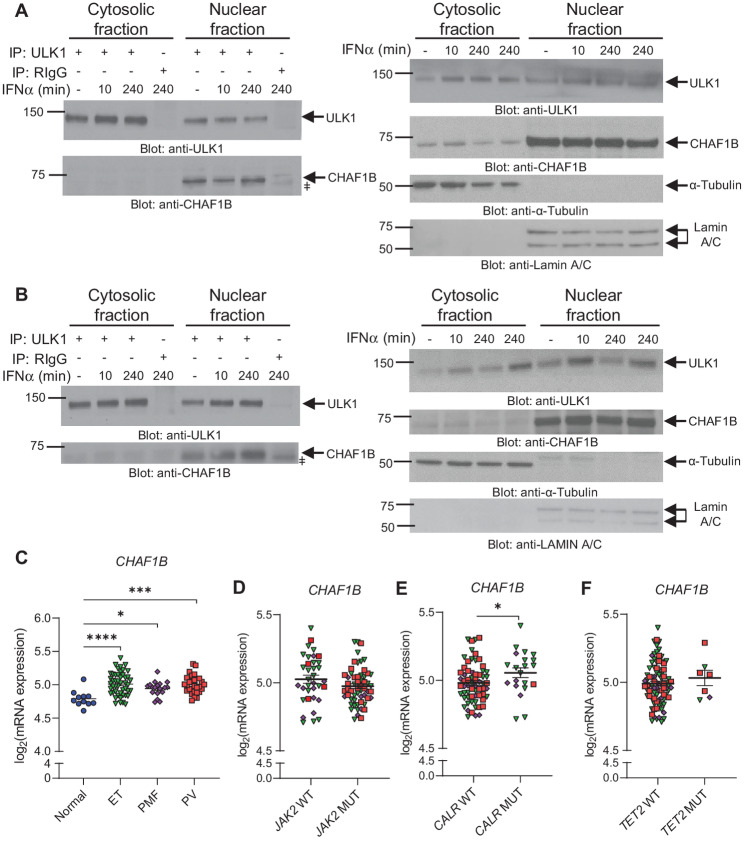
CHAF1B interacts with ULK1 in the nuclear compartment of *JAK2*^V617F^-positive cells and is overexpressed in patients with MPN. **A** and **B,** Left, ULK1–protein complexes were co-IP using an anti-ULK1 antibody from cytosolic and nuclear protein fractions isolated from untreated or IFNα-treated (10 or 240 minutes) HEL (**A**) or SET-2 (**B**) cells and then resolved by SDS-PAGE. As control, cytosolic and nuclear lysates isolated from cells treated with IFNα for 240 minutes were incubated with normal rabbit IgG (RIgG) antibody. Interaction between ULK1 and CHAF1B was assessed by immunoblotting with anti-ULK1 and anti-CHAF1B antibodies. Note: ǂ, unspecific band. **A** and **B,** Right, Equal amounts of cytosolic and nuclear protein lysates isolated from untreated and IFNα-treated HEL (**A**) and SET-2 (**B**) cells used for co-IPs were resolved by SDS-PAGE, transferred to PVDF membranes and then immunoblotted with antibodies against ULK1, CHAF1B, α-tubulin (cytosolic marker), and lamin A/C (nuclear marker), as indicated. **A** and **B,** Blots are representative of three independent experiments. **C,** Scatter dot plot of log_2_*CHAF1B* mRNA expression in neutrophils from healthy individuals (normal, *n* = 11) and patients with ET (*n* = 47), PMF (*n* = 18), and PV (*n* = 28). Data were extracted from NCBI GEO: GSE54646 study (21) and analyzed using GraphPad Prim 8. Shown are means ± SEM. Statistical analysis was performed using one-way ANOVA followed by Dunnett multiple comparisons test to assess *P* values between patients with MPN and healthy individuals. *, *P* < 0.05; ***, *P* < 0.001; ****, *P* < 0.0001. Scatter dot plots of log_2_*CHAF1B* mRNA expression in neutrophils from patients with ET (green triangles), PMF (purple diamonds), and PV (red squares) carrying wild-type (WT) or mutant (MUT) *JAK2* (**D**), *CALR* (**E**), and *TET2* (**F**) genes (*JAK2* WT: ET *n* = 20, PMF *n* = 10, PV *n* = 5; *JAK2* MUT: ET *n* = 26, PMF *n* = 8, PV *n* = 23; *CALR* WT: ET *n* = 27, PMF *n* = 11, PV *n* = 26; *CALR* MUT: ET *n* = 15, PMF *n* = 5, PV *n* = 1; *TET2* WT: ET *n* = 45, PMF *n* = 17, PV *n* = 24; *TET2* MUT: ET *n* = 2, PMF *n* = 1, PV *n* = 4). Data were extracted from NCBI GEO: GSE54646 study (21) and analyzed using GraphPad Prim 8. Patients for which no information was available for the mutational status for the *JAK2*, *CALR,* or *TET2* genes were excluded from the analysis. Shown are means ± SEM. Statistical analyses were performed using two-sample two-tailed *t* test: *, *P* < 0.05.

In previous studies, we had identified ULK1 as a key regulator of IFNα-inducible antineoplastic responses in MPN (15, 16). In further studies, we sought to identify novel regulators of IFNα-ULK1–driven responses in *JAK2*^V617F^-positive MPN-derived cells. As earlier reports have shown that ULK1 plays a role in both cytoplasmic and nuclear compartments (26, 27), we initiated these studies by performing co-IP of endogenous ULK1–protein complexes from cytoplasmic and nuclear fractions isolated from untreated and IFNα-treated *JAK2*^V617F^-positive HEL cells, followed by nLC/MS-MS analysis. We observed that ULK1 potentially interacts in the cytosolic fraction with 16 proteins in untreated cells, 63 proteins under both untreated and IFNα-treated (4 hours) conditions and with 14 proteins after 4 hours of IFNα treatment (Fig. 1A, cytosolic fraction). In the nuclear fraction, ULK1 was found to potentially bind 29 proteins under untreated conditions, 72 proteins under both untreated and IFNα-treated (4 hours) conditions and 41 proteins after 4 hours of IFNα treatment (Fig. 1A, nuclear fraction; ProteomeXchange Consortium dataset identifier: PXD037376). Moreover, overlap analysis of the 63 and 72 putative binding partners of ULK1 found under untreated and IFNα-treated conditions in the cytosolic and nuclear fractions, respectively, revealed eight proteins that could potentially interact with ULK1 in both compartments under these conditions (Fig. 1B, UT + IFNα). Gene ontology analyses were then performed for the 63 cytosolic and 72 nuclear putative ULK1-interacting proteins found under both untreated and IFNα-treated conditions. We found that ULK1 potentially binds proteins involved in several biological processes, including viral processes, mTOR signaling, and cell cycle (Fig. 1C; Supplementary Fig. S1). Interestingly, ULK1 was found to potentially bind CHAF1B both in the cytosolic and nuclear compartments (Fig. 1A and B), a protein involved in regulation of DNA metabolic process and part of the chromatin assembly complex (Fig. 1C; Supplementary Fig. S1). Notably, CHAF1B has been shown to be essential for normal hematopoiesis, and its overexpression has been reported to contribute to leukemogenesis (28). We next validated the binding between ULK1 and CHAF1B in untreated and IFNα-treated *JAK2*^V617F^-positive cells by co-IP followed by immunoblotting analyses. ULK1 specifically associated with CHAF1B in the nuclear compartment of both HEL and SET-2 cells (Fig. 2A and B). Importantly, using a previously published dataset (GEO accession: GSE54646; ref. 21), we found that the expression of *CHAF1B* is significantly upregulated in neutrophils isolated from patients with MPN with PV, ET, and PMF, when compared with neutrophils isolated from healthy donors (Fig. 2C). Interestingly, using the same dataset (GEO accession: GSE54646; ref. 21),when comparing the expression of *CHAF1B* in mutant versus wild-type cohorts for the *JAK2*, *CALR,* and *TET2* genes among patients with MPN (Fig. 2D–F), *CHAF1B* was found to be significantly higher in patients with MPN carrying a *CALR* mutation compared with patients carrying wild-type *CALR* (Fig. 2E). In this dataset (GEO accession: GSE54646), there was only information of 2 PMF patients carrying a *MPL* mutation, so no further analysis was performed for this gene.

### CHAF1B Negatively Regulates IFNα-inducible Transcription of ISGs

**FIGURE 3:**
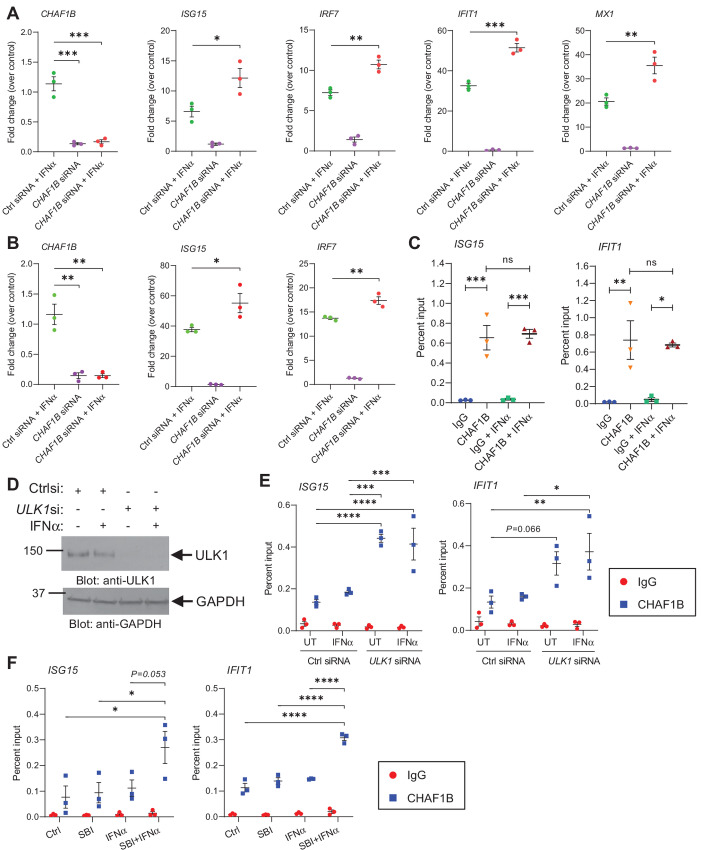
Gene-targeted inhibition of *CHAF1B* increases IFNα-inducible mRNA expression of ISGs in *JAK2*^V617F^-positive leukemic cells. qRT-PCR analyses of the indicated genes in control (Ctrl) siRNA or *CHAF1B* siRNA-transfected *JAK2*^V617F^-positive HEL (**A**) and SET-2 (**B**) cells, either left untreated or treated with IFNα for 6 hours. *GAPDH* mRNA expression was used for normalization. Data are expressed as fold change over control siRNA-transfected untreated cells (control) and represent means ± SEM of three independent experiments for each cell line. Statistical analyses were performed using one-way ANOVA followed by Tukey multiple comparisons test. *, *P* < 0.05; **, *P* < 0.01; ***, *P* < 0.001. **C,** ChIP assay was performed in untreated and IFNα-treated (for 6 hours at 5,000 IU/mL) HEL cells at the *ISG15* promoter and the *IFIT1* promoter for CHAF1B binding using an anti-CHAF1B antibody. IgG antibody was used for each promoter region as negative control. Scatter dot plots show data as percent enrichment relative to input ± SEM for three independent experiments. Statistical analyses were performed using one-way ANOVA followed by Tukey multiple comparisons test. ns, not significant; *, *P* < 0.05; **, *P* < 0.01; ***, *P* < 0.001. **D,** Immunoblot analysis of ULK1 expression in control siRNA (Ctrlsi) and *ULK1* siRNA (*ULK1*si) transfected HEL cells either left untreated or treated with IFNα (for 6 hours at 5,000 IU/mL), as indicated. GAPDH levels were used as loading control. Blots are representative of three independent experiments used to perform ChIP assays shown in **E**. **E,** ChIP assay was performed in untreated (UT) and IFNα-treated control (Ctrl) siRNA- and *ULK1* siRNA-transfected HEL cells at the *ISG15* promoter and the *IFIT1* promoter for CHAF1B binding using an anti-CHAF1B antibody. **F,** HEL cells were pretreated for 1 hour with either DMSO (Ctrl and IFNα groups) or SBI-0206965 (SBI; 10 μmol/L; SBI and SBI+IFNα groups) followed by 6 hours of treatment with either DMSO (Ctrl), SBI (10 μmol/L), IFNα (5,000 IU/mL) or SBI+IFNα, as indicated. ChIP assay was performed in HEL cells at the *ISG15* promoter and the *IFIT1* promoter for CHAF1B binding using an anti-CHAF1B antibody. **E** and **F,** IgG antibody was used for each promoter region as negative control. Scatter dot plots show data as percent enrichment relative to input ± SEM for three independent experiments. Statistical analyses were performed using two-way ANOVA followed by Tukey multiple comparisons test: *, *P* < 0.05; **, *P* < 0.01; ***, *P* < 0.001; ****, *P* < 0.0001. Relevant statistical differences are shown for the binding of CHAF1B to *ISG15* and *IFIT1* promoter regions between experimental conditions.

As CHAF1B has been shown to compete with transcription factors at specific DNA-binding sites impairing transcription of myeloid differentiation genes in leukemic cells (28), we next sought to determine whether CHAF1B plays a role in IFNα-dependent transcription of ISGs in *JAK2*^V617F^-positive cells. For this, we tested the effects of siRNA-mediated knockdown of *CHAF1B* on IFNα-induced transcription of ISGs in HEL and SET-2 cells using qRT-PCR analyses (Fig. 3A and B, left). Our results show that knockdown of *CHAF1B* increases IFNα-inducible mRNA expression of several ISGs (Fig. 3A and B). Moreover, through ChIP assays, CHAF1B was found to associate with the promoter regions of IFN-stimulated response element (ISRE) sites of *ISG15* and *IFIT1* (Fig. 3C). Thus, elevated CHAF1B expression in MPN cells may prevent the binding of transcription factors to the promoter region of ISGs, thereby impairing IFN responses. Next, to assess whether interaction of ULK1 with CHAF1B in the nucleus affects CHAF1B binding to ISGs’ promoter regions, we performed ChIP assays for CHAF1B using untreated and IFNα-treated control siRNA and *ULK1* siRNA-transfected HEL cells (Fig. 3D and E). Knockdown of ULK1 was found to increase the binding of CHAF1B to ISRE sites on *ISG15* and *IFIT1* (Fig. 3E). In addition, drug-targeted inhibition of ULK1 using SBI-0206965 (29) increased the binding of CHAF1B to the promoter region of *ISG15* and *IFIT1* when HEL cells were cotreated with IFNα (Fig. 3F). These results are consistent with our previous studies in which we show that ULK1 is required for increased transcription of ISGs (15, 16) and suggest that ULK1 prevents further binding of CHAF1B to ISGs’ promoter regions, facilitating the binding of IFN-activated transcription factors.

### CHAF1B Represses IFNα-dependent Antineoplastic Responses in MPN

**FIGURE 4:**
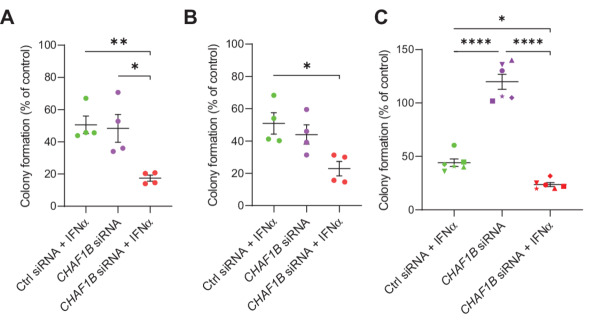
Inhibition of *CHAF1B* increases IFNα-induced anticlonogenic effects in MPN. HEL cells **(A**) and SET-2 cells (**B**) were transfected with either control (Ctrl) siRNA or *CHAF1B* siRNA, and leukemic CFU-L colony formation was assessed in clonogenic assays in the presence or absence of IFNα, as indicated. Data are expressed as percentage of colony formation over control siRNA-transfected untreated cells (control), and the scatter dot plots represent means ± SEM of four independent experiments for each cell line. **C,** Clonogenic capability of primary malignant erythroid progenitors isolated from PV patients transfected with either control (Ctrl) siRNA or *CHAF1B* siRNA and either left untreated or treated with IFNα, as indicated. Data are expressed as percentage of colony formation over control siRNA-transfected untreated cells (control) and shown are means ± SEM of six independent experiments using cells from six different patients with PV. Percentages for the same patient are represented by the same symbol and different patients have different symbols. **A–C,** Statistical analyses were performed using one-way ANOVA followed by Tukey multiple comparisons test. *, *P* < 0.05; **, *P* < 0.01; ****, *P* < 0.0001.

Next, to determine whether inhibition of CHAF1B affects IFNα-dependent antineoplastic responses, we performed colony assays evaluating the effects of siRNA-mediated knockdown of *CHAF1B* in HEL and SET-2 cells. IFNα treatment decreased the growth of colonies in HEL and SET-2 cells transfected with control siRNA, and this suppression was greatly increased in cells in which *CHAF1B* was knocked down (Fig. 4A and B). In further studies, we demonstrated that knockdown of *CHAF1B* was found to enhance the IFNα-induced antineoplastic effects against primary PV patient-derived progenitor cells in clonogenic assays (Fig. 4C). Together, our data suggest that the high levels of CHAF1B in MPN cells suppress the antitumor effects of IFNα.

## Discussion

Over the last decade, there have been substantial efforts to develop new therapeutic approaches for Philadelphia chromosome-negative myeloproliferative neoplasms and to ameliorate the complications from the disease and to prevent evolution into acute leukemia (8, 30, 31). The clinical development of pegylated forms of IFNα have resulted in easier to use and better tolerated regimens, with substantial efficacy (7, 9, 10, 32). However, some patients respond poorly to IFNα therapy (7, 9, 10, 32), underscoring the need for new therapeutic approaches to enhance IFN sensitivity and optimize clinical responses.

In previous work, we demonstrated that the kinase ULK1 is essential for the generation of IFN responses in MPN (15, 16). This prompted further studies to identify ULK1-interactive proteins that could be targeted therapeutically. Using mass spectrometry analysis screening, we identified CHAF1B as a novel interactor protein of ULK1 in the nuclear compartment of *JAK2*^V617F^-positive leukemic cells. CHAF1B is a component of the CAF-1 complex involved in the formation and loading of histone octamers during DNA replication (33, 34). Elevated CAF-1 correlates with a poor prognosis and decreased survival in numerous malignancies (35). In previous studies, we identified ULK1 activity in the cytoplasmic compartment as required for IFN responses, independent of its autophagy-related role (15, 16, 36). As overexpression of CHAF1B is a driver of leukemogenesis by blocking the binding of transcription factors to DNA sequences of myeloid differentiation genes (28), we decided to further investigate its role on IFNα-driven responses in MPN. We found that *CHAF1B* is overexpressed in neutrophils of patients with MPN as compared with healthy individuals. Inhibition of *CHAF1B* expression enhanced transcription of ISGs and IFNα-induced anticlonogenic effects against *JAK2*^V617F^-positive leukemic cells and primary PV patient cells. We show that these effects are, at least in part, mediated by the binding of CHAF1B to the promoter region of ISGs, potentially competing with the binding of IFNα-activated transcription factors. This finding raises the possibility that during normal hematopoiesis, CHAF1B acts as a feedback mechanism to balance IFN responses, allowing proper proliferation and differentiation of hematopoietic cells. In agreement, knockdown of *Chaf1b* and *Chaf1a* was shown to increase the accessibility to chromatin at specific enhancer sequences regulating differentiation of somatic cells in murine models (37). In another study, downregulation of these CAF-1 elements was shown to promote differentiation of myeloid stem and progenitor cells and the CAF-1 complex was found to control the access of ELF1 transcription factor to specific DNA-binding sites regulating cell lineage identity (38). Notably, siRNA-meditated inhibition of *ULK1* expression, as well as inhibition of ULK1 kinase activity, increased the binding of CHAF1B to the promoter region of ISGs in the presence of IFNα treatment. Hence, ULK1 kinase activity in the presence of IFNα stimulation and ULK1 interaction with CHAF1B prevent binding of CHAF1B to these regions promoting transcription of ISGs. These results are consistent with our previous study in which we show that genetic-targeted inhibition and deletion of *ULK1* decreases IFN-induced transcription of ISGs (15). These results suggest that ULK1/CHAF1B levels and complex formation/disintegration might act as a tight mechanism to control IFN responses in MPN. In other studies, expression of CHAF1B protein was found to correlate with the proliferation marker Ki-67 in several solid tumors, including breast, prostate, endometrial, cervical, thyroid, renal, gastric, pancreatic, and colon cancers (39, 40). In addition, elevated CHAF1B expression has been associated with advanced tumor grade and staging in melanoma, high-grade glioma, salivary gland tumors, prostate, endometrial, renal, and cervical cancers (40–44). Future studies are required to investigate whether CHAF1B interacts with ULK1 in these solid tumors and to further elucidate the potential biological role of this complex in various tumor types. Specifically, assessing whether inhibition of ULK1 affects CHAF1B's protumorigenic role in other tumor types may exhibit important clinical significance. In our study, we identify CHAF1B as a novel negative regulator of IFNα-mediated transcription of ISGs and, consequently, of the antineoplastic effects in MPN. These results support the future development of small-molecule drug-targeted inhibitors, antisense oligonucleotides or degraders (e.g., proteolysis targeting chimeras [PROTACs]) of CHAF1B for the treatment of patients with MPN, as an approach to enhance IFN sensitivity and overcome IFN resistance.

## Supplementary Material

Figure S1 Biological processes in which putative ULK1-protein complexes are involved in the cytosol.
