# Supplementary material for: Targeting CHAF1B Enhances IFN Activity against Myeloproliferative Neoplasm Cells
Source: Cancer Res Commun. 2023 May 31;3(5):943–51. doi: 10.1158/2767-9764.CRC-23-0010 (PMC10231401; doi:10.1158/2767-9764.CRC-23-0010)
Supplement: Figure S1 — Biological processes in which putative ULK1-protein complexes are involved in the cytosol. [file crc-23-0010-s01.pdf]

Supplementary Figures

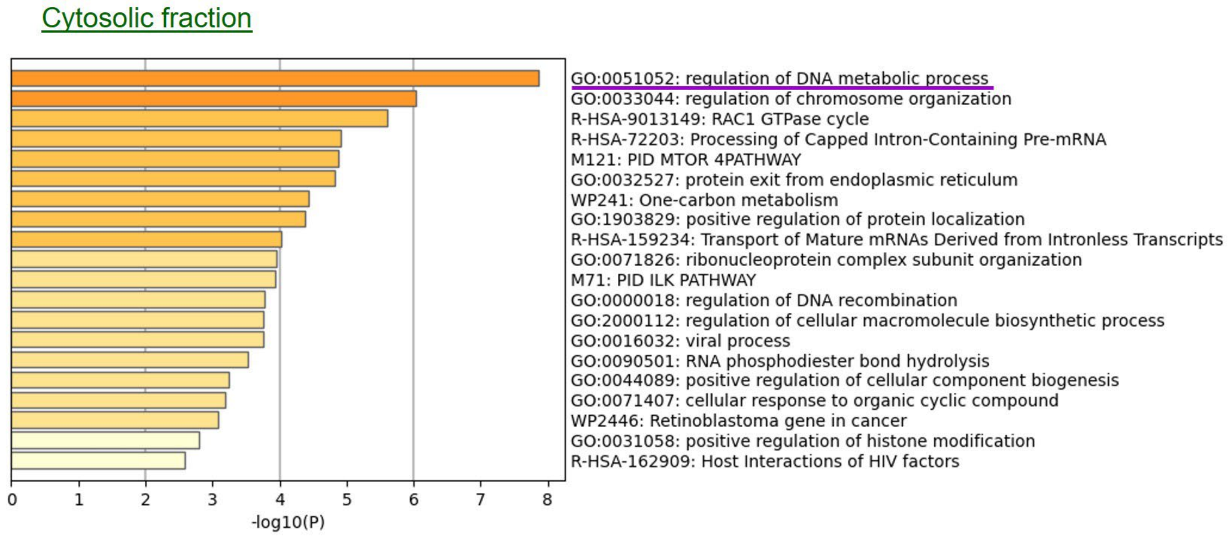

Supplementary Figure 1. Biological processes in which putative ULK1-protein complexes are involved in the cytosol. Gene ontology analyses of the 63 putative cytosolic ULK1 interacting proteins identified under both untreated and IFNα-treated conditions are shown. Underlined in purple are the biological events in which CHAF1B is involved.
